# Thermo-Economic Evaluation of Aqua-Ammonia Solar Absorption Air Conditioning System Integrated with Various Collector Types

**DOI:** 10.3390/e22101165

**Published:** 2020-10-16

**Authors:** Adil Al-Falahi, Falah Alobaid, Bernd Epple

**Affiliations:** Institut Energiesysteme und Energietechnik, Technische Universität Darmstadt, Otto-Berndt-Straße 2, 64287 Darmstadt, Germany; falah.alobaid@est.tu-darmstadt.de (F.A.); bernd.epple@est.tu-darmstadt.de (B.E.)

**Keywords:** solar cooling, absorption cycles, solar thermal collector, parabolic trough collectors, solar energy, ammonia

## Abstract

The main objective of this paper is to simulate solar absorption cooling systems that use ammonia mixture as a working fluid to produce cooling. In this study, we have considered different configurations based on the ammonia–water (NH_3_–H_2_O) cooling cycle depending on the solar thermal technology: Evacuated tube collectors (ETC) and parabolic trough (PTC) solar collectors. To compare the configurations we have performed the energy, exergy, and economic analysis. The effect of heat source temperature on the critical parameters such as coefficient of performance (COP) and exegetic efficiency has been investigated for each configuration. Furthermore, the required optimum area and associated cost for each collector type have been determined. The methodology is applied in a specific case study for a sports arena with a 700~800 kW total cooling load. Results reveal that (PTC/NH_3_-H_2_O)configuration gives lower design aspects and minimum rates of hourly costs (USD 11.3/h) while (ETC/NH_3_-H_2_O) configuration (USD 12.16/h). (ETC/NH_3_-H_2_O) gives lower thermo-economic product cost (USD 0.14/GJ). The cycle coefficient of performance (COP) (of 0.5).

## 1. Introduction

In recent years, the predominant technology in cooling systems worldwide is the conventional vapor compression cycle, which is known for its significant consumption of electricity due to the existence of a compressor and extremely high peak loads on the hottest summer days [1,2]. Solar energy is an attractive solution for operating air conditioning systems. It saves electricity and thus primary energy sources (natural gas or oil), and decreases the emission of air-polluting gases and greenhouse gases causing global warming. The air conditioning cooling loads and the available solar power match well with each other along the day and seasons. Therefore, solar air conditioning also leads to reducing the peak electric power demand, and thus reduces the initial cost used due to the expensive peak electricity [3]. According to the Internal Energy Agency (IEA), global energy demand will increase by 35% between 2010 and 2035 [4]. This increase is associated with world population and economic growth, especially in developing countries [5]. Heat-driven refrigeration cycles are a promising solution in this context. Absorption cycles are heat-driven cycles that can use solar energy directly without the need to transform it into electric energy. The other benefit of using the heat-driven cycle is the fact that the highest cooling demand is associated with the high solar energy in summer and that offers a good chance for the heat-driven cycle to match the cooling demand efficiently.

The temperature of the thermal energy source can affect the selection of the cooling technology as well as the performance of the cycle. The driving temperature depends on the solar energy capturing technology that can be adopted and efficiently available. Most of the cooling technologies are working in the range of 80–100 °C, which can be easily achieved using different low-cost solar energy collectors. Two kinds of absorption cycles are commercially available; depending on the refrigerant–absorbent pair; these two cycles are water–lithium bromide (H_2_O–LiBr) and ammonia–water (NH_3_–H_2_O) mixtures.

Kurem and Horuz [6] carried out a comprehensive study to investigate and analyze the absorption heat pump and using water–lithium bromide (H_2_O–LiBr) and ammonia–water (NH_3_–H_2_O) mixtures. The authors highlighted and pinpointed the advantages and disadvantages of both solutions. They concluded that the absorption heat transformer systems using water–lithium bromide solution produce better COP than that of using an ammonia–water solution.

Said et al. [7] presented an experimental investigation of a solar thermal powered ammonia–water absorption refrigeration system. The focus of this study lies in the design of the components of the absorption chiller, the ice storages, and the solar collector field as well as the integration of the data acquisition and control unit. The results of the experiments indicated a chiller coefficient of performance (COP) of 0.69 and a cooling capacity of 10.1 kW at 114/23/−2 (°C) representing the temperatures of the generator inlet, the condenser/absorber inlet, and the evaporator outlet respectively.

One of the advantages of absorption air-conditioning (ACC) cycles is the possibility of using several heat sources as input to the generator. Calise et al. [8] presented design of a novel solar-assisted combined cycle power plant. The system includes a solar loop equipped with a double-stage absorption chiller driven by high-temperature high-vacuum non-concentrating flat-plate solar thermal collectors. The simulation also includes a detailed thermo-economic model that accurately evaluates system capital and operating costs as a function of design and operating parameters. The simulation results show that a very high thermal efficiency of solar collectors—on average equal to 34%—is achieved. Results from the economic point of view were also satisfactory. Neyer et al. [9] analyzed the influence of different heat rejection units to a solar- or combined heat and power systems(CHP)-driven single-/half-effect ammonia/water absorption chiller. The simulation results are used to analyze the annual impact on the technical and economic performance of the new single-/half-effect concept in various configurations and boundary conditions. They concluded that application for solar- and CHP-driven systems are reaching non-renewable primary energy savings of 30–70% at almost equal costs compared to conventional systems.

Different types of solar thermal energy collectors can be used with single-effect absorption cycles. Aman et al. [10] developed a thermodynamic based on a 10 kW air-cooled ammonia–water absorption chiller driven by flat plate collector (FPC) for domestic application. Both energy and exergy analyses have been conducted to enhance the thermal performance of the cooling system. The analyses uncovered that the absorber is where the most exergy loss occurs (63%) followed by the generator (13%) and the condenser (11%). Bellos et al. [11] tested four solar collectors (evacuated tube collectors ETC, flat plate collectors FPC, parabolic through collectors PTC, and compound parabolic collectors CPC) in an absorption cooling system. A financial comparison between the four optimized systems proves that evacuated tube collectors are the most beneficial technology. On the other hand, a system with parabolic trough collectors is the exergetic optimum one, but its high capital cost renders it an unprofitable solution. Fong et al. [12], investigated a comparative study of different types of solar cooling systems for buildings in a subtropical city, which is commonly featured with long hot, and humid summer. The results show that solar electric compression refrigeration and solar absorption refrigeration had the highest energy saving and it will provide a promising application potential of solar cooling for buildings in the subtropical region.

In a recent study, Al-Falahi Adil [13] investigated the energy performance of a solar absorption air-conditioning system integrated with ETC, in the climate of Baghdad, Iraq. It was concluded that the seasonal collector efficiency and solar fractions are 54% and 58% respectively and absorption chiller COP was 0.44. Another study was presented by Galindo et al. [14], where they report experimental results of an absorption cooling system with a nominal capacity of 5 kW based on a parabolic trough collector, which operates with the ammonia-lithium nitrate mixture. The results of the parametric analysis revealed that the PTC field can provide up to 6.5 kW of useful heat to the absorption cooling system at temperatures up to 105 °C with thermal efficiencies up to 19.8% and exergy efficiency up to 14.93. The maximum value of the solar coefficient of performance reached 0.07. Cabrera et al. [15] presented a survey of the existing experiences and realizations on applications of PTC in solar cooling systems. as well as evaluated the use of the new collectors as an occasional alternative to other solar thermal collectors in air conditioning applications by dynamical simulation. The results for the case studies developed in this work show that PTC presents similar levelized costs of energy for cooling than flat plate collectors (FPC) and lower than evacuated tube collectors (ETC) and compound parabolic collectors (CPC).

Jakob et al. [16], presented a design of an air conditioning by diffusion absorption using the mixture NH_3_-H_2_O, with a cooling capacity of 2.5 kW. The unit was built and tested, achieving a maximum COP of 0.38. The collector used was an indirectly coupled flat plate collector (FPC).

Parabolic trough collectors are frequently employed for a solar steam generation because relatively high temperatures can be obtained without serious degradation in the collector efficiency. Qu et al. [17] developed a model of solar absorption cooling and heating system driven by PTC using transient system simulation software. The system incorporates 52 m^2^ of parabolic trough solar collectors; a 16 kW absorption chiller capacity. The performances of the system have been analyzed and validated with experimental data under various solar radiations and wind velocity. They concluded that the absorption air conditioning (AAC) cycle may potentially provide 20% of the heating and 39% of the cooling demands of the building. Tzivanidis and Bellos [18] developed a numerical model is in order to simulate the dynamic performance of a solar cooling system. Many parameters have been investigated through sensitivity analyses and their optimum values are determined. The results proved that by using a PTC module with an aperture area of 14 m^2^, a building area of about 25 m^2^ can be cooled for 13 h daily during the summer period. The optimum specific mass flow rate was determined to 0.03 kg/s m^2^ and the optimum storage tank volume to 0.3 m^3^. Moreover, they presented a case study for a typical building of 100 m^2^ with very satisfying results, where four PTC modules are used in parallel connection.

Molero-Villar et al. [19] developed a complete solar thermal cooling system using TRNSYS model to compare a configuration with just hot storage (of typical capacity 40 L/m^2^ of solar collector surface) and a configuration with both, hot and cool storages. In their study, they analyzed the effect of other variables on the optimal configuration: collector efficiency curve, COP of the absorption chiller, storage size, and temperature set-points of the chillers. They concluded that the most suitable configuration is very sensible to the solar collector area. As the collector area increases, the advantages of cool storage vanish. Increasing the collector area tends to increase the temperature of the hot storage, leading to higher thermal losses in both the collector and the tank. When the storage volume is concentrated in one tank, these effects are mitigated.

Said [20] demonstrated and developed an alternate design for a 24 h operating solar-powered absorption refrigeration technology. The development includes an in-depth review of the design and operation of the conventional and solar-assisted absorption refrigeration systems coming-up with new alternative designs, detailed thermodynamic analysis of some of the new alternative designs, and selection of the most suitable alternative design. The analysis indicates that continuously operating a solar-powered ammonia–water absorption system with refrigerant storage is the most suitable alternative design for an uninterrupted supply of cooling effect.

Abdelhay et al. [21] carried out a parametric study to investigate the effects of the different design and operating parameters on the polygeneration plant energetic and exergetic parameters. Results reveal that the highest exergy loss was found to occur in PTC solar field with about 82.42% of the plant exergy destruction and for the cooling part to balance between the COP of the absorption refrigeration system and PTC area, the generator temperature should be 85–90 °C. Moreover, an economic evaluation of the proposed integrated system showed the unit cooling price (0.003 $/kW hr) and the highest exergetic efficiency is (23.95%). The absorption chiller capacity was 3.6TR (Ton of Refrigeration). In the same regard, Leiva et al. [22] performed a comparison between the levelized cost and thermo-economic methodologies of a solar polygeneration plant. They concluded that through the study they found differences between the two methods and this represent an increase of about 35.1% in the case of the electricity, and a reduction in the cost associated to the water, cooling, and heat production by around 34.4%, 78.1%, and 97.6%, respectively.

Related to thermo-economics, Al-Falahi [23] presented the design and thermo-economic evaluation of absorption air conditioning (AAC) to compare two various collectors (PTC, ETC). The results reveal that parabolic trough collectors combined with H_2_O–LiBr (PTC/H_2_O–LiBr)gives lower design aspects and minimum rates of hourly costs (USD 5.2/h), while ETC/H_2_O–LiBr configuration give USD 5.6/h. The H_2_O–LiBr thermo-economic product cost is USD 0.14/GJ. The cycle coefficient of performance COP was in the range of 0.5 to 0.9. Salehi [24] studied the feasibility of solar assisted absorption heat pumps for space heating; in this study, single-effect LiBr/H_2_O and NH_3_/H_2_O absorption and absorption compression-assisted heat pumps are analyzed for heating loads of 50–2 MW(MegaWatt). Utilizing geothermal hot springs as heat sources for refrigerant evaporation. the results show that the maximum achievable exergy efficiency for the NH_3_-H_2_O and LiBr-H_2_O absorption systems is 0.23 and 0.25, respectively. while, the solar heating system has the highest product unit cost, mainly due to its high capital costs. The minimum obtainable product unit costs for the NH3/H_2_O and LiBr/H_2_O absorption systems are about 22 $/GJ and 55 $/GJ, respectively.

Marc [25] studied an experimental solar absorption cooling. The solar field consists of 36 double-glazed flat-plate solar collectors of a total aperture area of 90 m^2^ coupled to a single-effect absorption chiller with cooling capacity at 30 kW at 90/30/11 (°C) representing the temperatures of the generator, the condenser, and the evaporator respectively. The maximum electrical coefficient of performance was 1.65. Zhai [26] used the evacuated tube solar field for 8 kW cooling capacity. The system consumed and amount of 92 m^2^ of solar area. Lopez et al. [27] performed energy and exergy analysis to compare several system cooling cycles configurations and different solar thermal technologies: evacuated tube, parabolic trough, and linear Fresnel solar collectors. The results show that NH_3_-H_2_O/(ETC or PTC) combinations offer the best efficiencies. In their work ammonia-based working fluid mixture is considered.

It is clear from the literature that solar energy has a great influence on refrigeration and/or air conditioning processes. Different types and configurations of solar collectors have been applied for such purposes. The most commonly used was the evacuated tube and parabolic through collectors especially for large capacities. Moreover, it has been noticed that H_2_O-LiBr and NH_3_-H_2_O have been used for most of the research activities in this regard for air conditioning applications. The novelty of this work emerged from the connection type between the solar field and the absorption chiller. A flash evaporation tank has been used as an intermediate unit between both parts to generate steam; known as direct or in situ concept system, in which two-phase flow is allowed in the collector receiver so that steam is generated directly. The sub-cooled water is heated to boiling and steam forms directly in the receiver tube. The system use water, a superior heat transport fluid. The flash system uses a sensible heat change in the working fluid, which makes the temperature differential across the collector relatively high. The rapid increase in water vapor pressure with temperature requires a corresponding increase in system operating pressure to prevent boiling. The main objective of this work is to optimize and design NH_3_-H_2_O absorption air conditioning systems that are operated by different types of solar collectors (ETC and PTC), based on energy, exergy, design, and cost. Selecting the best operating condition is also been the focus. The following general outlines are proposed for this work:

✓ NH_3_-H_2_O absorption cycles have been studied under different operating conditions. The selection was made based on the best-operating conditions.
✓ Two different types of solar collectors (ETC and PTC) have been compared while combining them with absorption cycles.
✓ A detailed mathematical model has been performed.
✓ The comparison is performed based on the terms of energy, exergy, design, cost, and thermo-economic. The design technique of modeling has been adopted in this study.
✓ Based on the optimized selection, a case study for 700–800 kW (200–230 TR) cooling load has been performed.

## 2. Thermal Solar Cooling System Description

**Figure 1:**
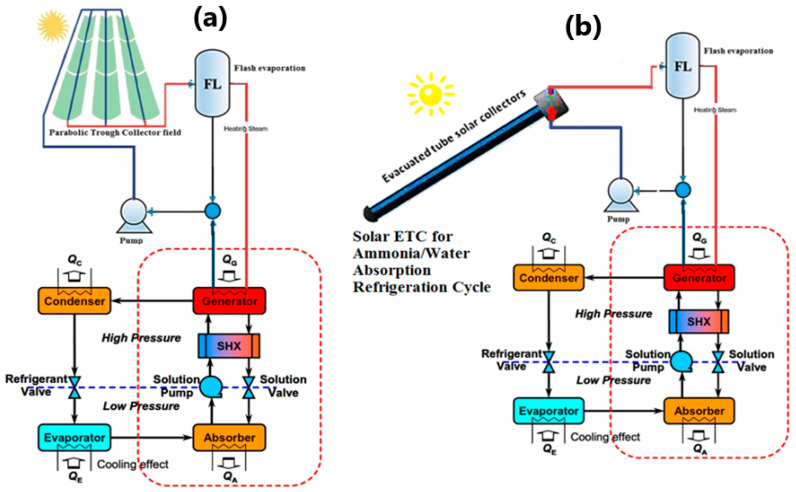
(**a**) Parabolic trough (**b**) evacuated tube solar collectors assisted NH_3_-H_2_O absorption air conditioning cycle.

The working principle of the ammonia–water absorption refrigeration system is based on the simple vapor absorption refrigeration system. In this system ammonia is used as the refrigerant and water is used as the absorbent. The ammonia–water absorption system is used in the domestic as well the commercial applications where the requirement of the temperature is above 0 °C. The major advantage of the ammonia–water solution is that water has a strong affinity for ammonia and they are soluble with each other in wide operating conditions that occur in different refrigeration applications. Further, the ammonia–water solution is highly stable and works well with many materials except copper and its alloys that get corroded in the presence of ammonia. The proposed system, Figure 1a,b, consists of two subsystems, namely: (i) the solar field, and (ii) the absorption cycle. The solar field consists of the following:

➢ Evacuated tube collector (ETC) and/or parabolic trough collector (PTC) for thermal power generation. Water working fluid is the main fluid through the solar part.
➢ A Flash evaporation tank is used to separate steam at lower pressure. This is a vertical flash vessel, with the inlet of high-pressure high-temperature water located at about one-third of the way up to its height. The standard design of flash vessels requires that the diameter of the vessel is chosen so that the steam flows toward the top outlet connection at no more than about 3 m/s. This should ensure that any water droplets could fall through the steam in a contra-flow, to the bottom of the vessel. Adequate height above the inlet is necessary to ensure separation. The separation is also facilitated by having the inlet projecting downward into the vessel. The water connection is sized to minimize the pressure drop from the vessel to the pump inlet to avoid cavitation.
➢ The pumping unit has been used for flow circulation and pressure drop issues.

The absorption refrigeration cycle (NH_3_-H_2_O) consists of an absorber, a generator, a condenser, an evaporator, expansion valves, a solution heat exchanger (SHX), and a solution pump as illustrated in Figure 1.

➢ In the evaporator, low-pressure liquid-vapor mixture refrigerant pure ammonia (NH_3_), evaporates on the outer surface of the evaporator’s tube bundle. Heat is supplied to the evaporator by water flowing inside the evaporator’s tubes; this chilled water is subsequently used for refrigeration. The refrigerant vapor leaving the evaporator as saturated vapor enters the absorber at low-temperature and low-pressure conditions.
➢ In the absorber, The refrigerant vapor is then absorbed and dissolved by the weak ammonia–water (NH_3_-H_2_O) solution coming from the generator. It is worth mentioning that the weak solution is the solution with less amount of refrigerant, i.e., highly concentrated by water. The heat liberated during the absorption process is removed by using cooling water flowing inside the tubes.
➢ The solution pump receives a strong NH_3_-H_2_O solution and delivers it at high pressure to the generator via the solution heat exchanger. Note that the solution is a saturated liquid and hence the pump work is very small.
➢ In the generator, the high-pressure solution entering the generator is heated and the ammonia in the solution is vaporized. The heat for this process could be provided by solar energy or a gas burner. The remaining weak NH_3_-H_2_O solution exits via the heat exchanger to the absorber through a pressure-reducing solution valve.
➢ A solution heat exchanger (SHX) is included here; as the solution after the pump requires heating and the hot solution leaves the generator would increase the absorber temperature if it were not cooled before entry.
➢ In the condenser, the high-pressure, high-temperature refrigerant vapor leaving the generator is passed to the condenser where it is cooled by cooling water. The refrigerant saturated vapor is condensed to a liquid. This liquid is then flowed through an expansion refrigerant valve and to the evaporator.

The cycle is considered a closed cycle related to the working flowed through it.

## 3. Methodology, Assumptions and Mathematical Model

The proposed configurations in this work are required iterative programming in order to calculate the complicated streams (recycle and backward streams). Hence; to develop a specific model for this innovative kind of plan, the Simulink software, a product of a MATLAB work, has been chosen for its high versatility and capacity to handle unsteady situations. Two models were built according to the proposed configurations (ETC/NH_3_-H_2_O and PTC/NH_3_-H_2_O) and the design calculation method. The system border streams (outlet temperature, ambient temperature, inlet cooling water temperature, etc.) are assigned by the user than the entire design data (area, length, volume, mass flow rate, etc.) Will then be calculated. Therefore; a user would assign the amount of needed cooling load on the evaporator then all possible data for all the system units would be calculated in sequence. Specifying the system cooling load would calculate the required thermal load. Besides; the required design limits and performance calculations would be pass out instantly. The design operating conditions and assumptions that have been considered in this work are listed in Table 1.

Saturated liquid and vapor phases of pressure, temperature, enthalpy, specific volume, and specific entropy are recorded after the modeled blocks and lookup tables. The source of physical properties is obtained by the chemistry book on the National Institute of Standards and Technology NIST website [28]. Generally, the optimization process has been done in order to bring down costs and techno-economic solutions.

### 3.1. Thermodynamic Model

A schematic diagram of the main component of the proposed is shown in Figure 1. The mathematical approaches used in the analysis for solar absorption air conditioning (AAC) plants are performed according to the 1st and 2nd laws of thermodynamics. The following assumptions are considered in the developed mathematical model of the system:

(1) The system runs in a steady-state condition.
(2) Heat losses to the surroundings are neglected.

The thermodynamic model of each component is explained in the following section.

#### 3.1.1. ETC Model

For ETC, the solar collector instantaneous efficiency can be determined from its characteristic curve using solar irradiance, mean collector, and ambient temperatures. The curve used for FPC is expressed by Equation (1) [29,30]:

(1) $$\eta_{etc}=\eta_{o}-a_{1}\left(\frac{T_{o}-T_{amb}}{I_{s}}\right)-a_{2}{\left(\frac{T_{o}-T_{amb}}{I_{s}}\right)}^{2}.I_{s}$$

where $\eta_{etc}$ is the ETC efficiency, $\eta_{o}=0.665$, $a_{1}$ = 2.9 W/m^2^. °C, $a_{2}$ = 0.0019 W/m^2^. °C^2^, $I_{s}$ is the solar flux over the collector area (W/m^2^), $T_{o}$ is the outlet collector top temperature (°C), and ambient temperature $T_{amb}$ in (°C).The useful collector thermal load $Q_{th}$ (kW), may exist according to the following relation;

(2) $$Q_{th}=M_{col}^{.}\times C_{p}\times \left(T_{o}-T_{i}\right)$$

where, $M_{col}^{.}$ in kg/s, $C_{p}$ in kJ/kg °C and the temperature difference is in °C.

The collector total area $A_{t}$ (m^2^), is estimated based on the collector energy balance equation as a function of collector efficiencies using;

(3) $$A_{t}=\frac{Q_{th}}{\eta_{etc}\times I_{s}}$$

As shown in Equation (3), if the value of the heat exiting the collector is fixed, the necessary collector area is inversely proportional to solar radiation. This is why the value of solar radiation is fundamental for the development of projects involving solar energy. The ETC module aperture area $A_{etc}$, m^2^ can be calculated as;

(4) $$A_{etc}=D_{t}\times L_{t}\times NOT$$

where, $D_{t}$ is the tube diameter(m), $L_{t}$ is the tube length(m), and NOT is the number of tubes per one module. The total number of evacuated tube collectors NOC can be calculated from the following equation.

(5) $$NOC=\frac{A_{t}}{A_{etc}}$$

The number of loops NOL, loop area $A_{loop}$, and the loop length, $L_{loop}$ can be calculated by assigning the hydraulic mass flow rate $M_{hyd}^{.}$, kg/s. $M_{col}^{.}$ will be calculated based on the load between the flash tank and the AAC unit:

(6) $$NOL=\frac{M_{col}^{.}}{M_{hyd}^{.}}$$

(7) $$A_{loop}=\frac{A_{t}}{NOL}$$

(8) $$L_{loop}=\frac{A_{loop}}{L_{t}}$$

#### 3.1.2. PTC Model

The corresponding efficiency equation for the medium-high temperature parabolic trough collectors (PTC) is given in Equation (9) [30]:

(9) $$\eta_{ptc}=\eta_{o}-a_{11}\left(T_{o}-T_{amb}\right)-a_{21}\left(\frac{T_{o}-T_{amb}}{I_{s}}\right)-a_{31}{\left(\frac{T_{o}-T_{amb}}{I_{s}}\right)}^{2}$$

where, $a_{11}$ = 4.5 × 10^−6^ 1/°C, $a_{21}$ = 0.039 W/m^2^. °C, $a_{31}$ = 3 × 10^−4^ W^2^/m^2^. °C^2^, $\eta_{o}=0.75$.

The thermal efficiency of a PTC is the captured or gained useful energy divided by the incident radiation on the aperture plane, as shown in Equation (10)

(10) $$\eta_{PTC}=\frac{Q_{u}}{A_{PTC}\times I_{s}}$$

where $\eta_{PTC}$ is the thermal efficiency of the collector; Qu is the useful power (W); $A_{PTC}$ is the aperture area of the collector (m^2^); and I is the solar radiation absorbed in the aperture plane (W/m^2^).

The modification of Equation (10) yields Equation (11):

(11) $$A_{PTC}=\frac{Q_{u}}{\eta_{PTC}\times I_{s}}$$

However, the required land area for the installation of solar collectors is higher than the aperture area of collectors. This value varies approximately between three to four times the aperture area of collectors. The land area is greater because there is a distance between collectors besides the required space for pipes and other accessories of the system. The collector useful energy equation may exist according to the following relation:

(12) $$Q_{u}=M_{col}^{.}\times \Delta h_{o-i}$$

where Δ*h* is the enthalpy difference across the collector in kJ/kg, and $M_{col}^{.}$ is the total field mass flow rate in kg/s. The PTC total length $L_{PTC}$ is then calculated based on collector width $W_{c}$ (m) and glass envelope diameter $D_{env}$ (m):

(13) $$L_{PTC}=\frac{A_{PTC}}{W_{c}-D_{env}}$$

By knowing the total mass flow rate, which is calculated from the boiler heat exchanger load, and by assigning the hydraulic mass flow rate as an input, the total number of loops $N_{loop}$, loop area $A_{loop}$, loop width $W_{loop}$, and the number of solar PTC’s ($N_{PTC^{\prime }s})$ are then calculated as follows;

(14) $$N_{loop}=\frac{M_{col}^{.}}{M_{hyd}^{.}}$$

(15) $$A_{loop}=\frac{A_{PTC}}{N_{loop}}$$

(16) $$W_{loop}=\frac{A_{loop}}{L_{m}}$$

where $L_{m}$ (m) is assigned as module length.

(17) $$N_{PTC^{\prime }s}=\frac{A_{PTC}}{L_{m}\left(W_{c}-D_{env}\right)}$$

Total pressure losses $P_{tloss}$ are calculated based on major and minor losses along the field length. The general loss equation is performed as following [31,32]:

(18) $$P_{tloss}=N_{loop}\times \Delta P_{loop}$$

where

(19) $$\Delta P_{loop}=\frac{32\times f\times L_{loop}\times M_{hyd}^{.}{}^{2}}{\rho \times \pi^{2}\times D_{t}}$$

$D_{t}$ is the inner tube diameter(m)

(20) $$f={\left[\left(1.82\times \log Re\right)-1.64\right]}^{-2}$$

(21) $$Re=4\times \frac{M_{hyd}^{.}}{\mu \times \pi \times D_{t}}$$

#### 3.1.3. Flashing Tank

Flash Cyclone tank design data are calculated as follows:

Tube Inlet and outlet tank steam area $A_{t_{i}}$ based on steam velocity $V_{st}$, m/s, and vapor density $\rho_{v}$, kg/m^3^:

(22) $$A_{t_{i}}=\frac{M_{st}}{\rho_{v}\times V_{st}}$$

Tube diameter $D_{t}$ (m):

(23) $$D_{t}={\left(\frac{A_{t_{i}}\times 4}{\pi }\right)}^{0.5}$$

Flash tank height $H_{fst}$ (m) [31]:

(24) $$H_{fst}=7.15\times D_{t}$$

Flash tank width $W_{fst}$ (m):

(25) $$W_{fst}=3.5\times D_{t}$$

Flash tank total volume $Vol_{fst}$ (m^3^):

(26) $$Vol_{fst}=\left(\frac{pi}{4}\right)\times W_{fst}{}^{2}\times H_{fst}$$

Flashing enthalpy $h_{fsh}$ is equal to the enthalpy coming from the solar collector $h_{col}$, kJ/kg:

(27) $$h_{fsh}=h_{col}$$

The flashing dryness fraction $X_{fsh}$ is calculated based on flashing enthalpy $h_{fsh}$, liquid enthalpy $h_{f}$, (kJ/kg) and dry vapor enthalpy $h_{g}$ (kJ/kg):

(28) $$X_{fsh}=\frac{h_{fsh}-h_{f}}{h_{g}-h_{f}}$$

The total mass flow rate $M_{total}$ (kg/s), and unvaporized water $M_{w}$ (kg/s) are given by Equations (29) and (60), respectively:

(29) $$M_{total}=\frac{M_{st}}{X_{fsh}}$$

(30) $$M_{w}=\left(1-X_{fsh}\right)\times M_{total}$$

#### 3.1.4. Pump

The pump work $W_{p}$, kW can be calculated using [33]:

(31) $$W_{p}=M_{total}\times \frac{\Delta P}{\rho }\times \eta_{p}$$

where ΔP, bar is the total pressure difference and it is calculated as:

(32) $$\Delta P=P_{high}+P_{loss}$$

Outlet pump enthalpy $h_{po}$ (kJ/kg):

(33) $$h_{po}=\left(\frac{W_{p}}{M_{total}}\right)+h_{pi}$$

#### 3.1.5. Mathmatical Model of the Absorption Cycle

A schematic diagram of a single-effect NH_3_-H_2_O absorption system is shown in Figure 1. The following assumptions are considered in the developed mathematical model of the absorption cycle:

(1) Refrigerant leaving the evaporator is saturated ammonia vapor.
(2) Refrigerant leaving the condenser is saturated ammonia liquid.
(3) No liquid carry over from evaporator.
(4) Refrigerant vapor leaving the generator has the equilibrium temperatures of the weak solution of the generator’s pressure and is salt free.
(5) The solutions leaving the generator and absorber are saturated.
(6) The pumping process is isentropic.

The thermodynamic model of each component is explained in the following section.

The mathematical model for determining the thermal powers to be transferred in the main components of the absorption cooling system, was a first-law thermodynamic model, which was based on the Equations (34) to (62).

##### Absorber

In the absorber, the saturated vapor refrigerant from evaporator is absorbed by the weak solution from generator and converted to low pressure strong solution. The generated heat is removed by the cooling water. For the absorber, the flow factor parameter is especially important in the calculation procedures of the thermal power through the model. The flow rate ratio factor f is calculated based on the absorber temperature as following [33]:

(34) $$f=0.4067\times exp\left(0.05606\times T_{a}\right)+5.09e-07\times exp\left(0.3293\times T_{a}\right)$$

The strong solution mass flow rate $M_{str}$ (kg/s) is calculated based on the total refrigerant flow rate $M_{r}$ (kg/s) and the flow factor f:

(35) $$M_{str}=M_{r}\times f$$

The weak solution mass flow rate $M_{wk}$ (kg/s):

(36) $$M_{wk}=M_{str}-M_{r}$$

The Absorber thermal power $Q_{a}$, kW is calculated based on the energy balance across the absorber between inlet and outlet streams, where *H* denotes to enthalpy(kJ/kg):

(37) $$Q_{a}=M_{r}\times \left(H_{e-abs}+\left(M_{wk}\times H_{hex-a}\right)-\left(M_{str}\times H_{a-hex}\right)\right)$$

Overall heat loss $U_{a}$ (kW/m^2^ °C) [34]:

(38) $$U_{a}=1.6175+0.1537e-3\times T_{a}+0.1825e-3\times T_{a}^{2}-8.026e-8\times T_{a}^{3}$$

The absorber area $A_{a}$ (m^2^):

(39) $$A_{a}=\frac{Q_{a}}{U_{a}\times \Delta T}$$

##### Solution Heat Exchanger SHX

A solution heat exchangeris (SHX) is included in the absorption cycle to exchange heat between the streams flowing in and out of the generator, the use of the SHX augments the coefficient of performance (COP) by reducing the heat released in the absorber and the heat supplied to the generator. For the heat exchanger unit, the mass flow ratio is obtained as follows f:

(40) $$f=\frac{M_{str}}{M_{str}-M_{wk}}$$

The NH_3_ concentration percentage XNH3 [33,35]:

(41) $$XNH3=\frac{\frac{M_{str}}{f}}{M_{r}+M_{wk}}$$

The outlet heat exchanger stream temperature towards the absorber unit $T_{hex\_a}$ (°C) is calculated based on the heat exchanger effectiveness $\varepsilon_{hex}$:

(42) $$T_{hex\_a}=T_{g}-\left(\varepsilon_{hex}\times \left(T_{g}-T_{a}\right)\right)$$

Outlet heat exchanger temperature to the generator unit $T_{hex\_g}$ (°C):

(43) $$T_{hex\_g}=T_{a}+\left(\varepsilon_{hex}\times \left(T_{g}-T_{a}\right)\right)$$

The enthalpy of NH_3_-H_2_O solution outlet to the absorber unit $H_{hex\_a}$ (kJ/kg) is calculated based on temperature $T_{hex-a}$ (K) and specific heat capacity $Cp_{NH3}$ (kJ/kg K):

(44) $$H_{hex\_a}=Cp_{NH3}\left(T_{hex-a}+273\right)\times \left(T_{hex-a}+273\right)$$

where the specific heat capacity for NH_3_, $Cp_{NH3}$ (kJ/kg K):

(45) $$Cp_{NH3}=\frac{27.31+0.02383\times T+1.707e-5\times \left(T^{2}\right)-1.185e-8\times \left(T^{3}\right)}{17.0305}$$

The enthalpy of NH_3_-H_2_O solution outlet to the generator unit $H_{hex\_g}$ (kJ/kg):

(46) $$H_{hex\_g}=Cp_{NH3}\left(T_{hex-g}+273\right)\times \left(T_{hex-g}+273\right)$$

The heat exchanger thermal power $Q_{hex}$ (kW) is then calculated based on the thermal energy balance between inlet and outlet streams:

(47) $$Q_{hex}=\left(M_{str}-M_{wk}\right)\times \left(f-1\right)\times \left(H_{g-hex}-H_{hex-a}\right)$$

Enthalpy stream from the absorber towards the heat exchanger $H_{a-hex}$ (kJ/kg):

(48) $$H_{a-hex}=H_{hex-g}-\left(\left(\frac{f-1}{f}\right)\times \left(H_{g-hex}-H_{hex-a}\right)\right)$$

Mean temperature $T_{hex_{m}}$ (°C):

(49) $$T_{hex_{m}}=\frac{T_{hex-a}+T_{hex-g}}{2}$$

Overall heat loss $U_{hex}$ (kW/m^2^ °C):

(50) $$U_{hex}=1.6175+0.1537e-3\times T_{hex_{m}}^{1}+0.1825e-3\times T_{hex_{m}}^{2}-8.026e-8\times T_{hex_{m}}^{3}$$

Heat exchanger area $A_{hex}$ (m^2^):

(51) $$A_{hex}=\frac{Q_{hex}}{U_{hex}\times \Delta T}$$

##### Generator

The strong solution is introduced to the generator, where heat energy is supplied by hot water or steam. The heat energy separates the refrigerant vapor from the solution and a high water concentrated solution is exits to HEX. The outlet enthalpy to the HEX, $H_{g\_hex}$ (kJ/kg) is calculated based on the energy balance between both units and flow rate ratio [34,35,36]:

(52) $$H_{g\_hex}=\frac{\left(\frac{Q_{g}}{M_{r}}\right)-H_{g-cond}+\left(f\times H_{hex-g}\right)}{f-1}$$

Overall heat loss $U_{g}$ (kW/m^2^ °C) through the generator tubes is calculated as following [36]:

(53) $$U_{g}=1.6175+0.1537e-3\times T_{g}^{1}+0.1825e-3\times T_{g}^{2}-8.026e-8\times T_{g}^{3}$$

Generator area $A_{g}$ (m^2^):

(54) $$A_{g}=\frac{Q_{g}}{U_{g}\times \Delta T}$$

Inlet driving steam mass flow rate Ms, (kg/s) is calculated based on the latent heat $h_{fg}$, (kJ/kg) from the heat source:

(55) $$Ms=\frac{Q_{g}}{0.95\times h_{fg}}$$

where $h_{fg}$ is the latent heat of distillate vapor evaporation (pure ammonia) [33,34,35,36]:

(56) $$h_{fg}=-46.53\times exp\left(0.02096\times T\right)+1305\times exp\left(-0.001835\times T\right)$$

##### Condenser

In the condenser, the ammonia vapor is liquefied by means of the cooling water supplied by the auxiliary cooling system. Once the ammonia has been condensed. The condenser thermal power $Q_{c}$ (Kw) can be calculated using:

(57) $$Q_{c}=M_{r}\times \left(H_{g-cond}-H_{cond-e}\right)$$

The overall heat loss $U_{c}$ (kW/m^2^ °C):

(58) $$U_{c}=1.6175+0.1537e-3\times T_{c}^{1}+0.1825e-3\times T_{c}^{2}-8.026e-8\times T_{c}^{3}$$

The condenser area $A_{c}$, (m^2^):

(59) $$A_{c}=\frac{Q_{c}}{U_{c}\times \Delta T}$$

Inlet cooling water enthalpy $H_{c_{cwi}}$ (kJ/kg):

(60) $$H_{c_{cwi}}=421.2\times exp\left(0.004008\times T_{c_{cwi}}\right)-435.9\times exp\left(-0.007559\times T_{c_{cwi}}\right)$$

Outlet cooling water enthalpy $H_{c_{cwo}}$ (kJ/kg):

(61) $$H_{c_{cwo}}=\left(\frac{Q_{c}}{M_{cw}}\right)+H_{c_{cwi}}$$

##### Evaporator

In the evaporator, the low-pressure, low temperature refrigerant (after the refrigerant expansion valve) is sprayed over the evaporator tube, where the cold water circulates through the tubes and gets chilled. The refrigerant mass flow rate $M_{r}$ (kg/s) can be calculated based on the energy balance across the evaporator [36]:

(62) $$M_{r}=\frac{Q_{e}}{H_{e-abs}-H_{cond-e}}$$

where $Q_{e}$ is a thermal load on evaporator unit, (kW).

#### 3.1.6. Availability, Cost, and Performance 

The availability, cost, and performance were calculated based on the formulas provided in the references [37,38,39,40,41]. The availability equation for any system goes under a steady-state, uniform flow process can be developed with the first and second law of thermodynamics. Neglecting the changes in kinetic and potential energy. The general form of the availability can be defined by the following equation [37]:

(63) $$A_{2}-A_{1}=A_{q}+A_{w}+A_{fi}-A_{fo}-I$$

where $A_{2}-A_{1}=0$ is the non-flow availability change in a steady-state condition, $A_{q}=\sum_{J}\left(1-T_{amb}/T_{J}\right)Q_{J}$ is the availability transfer due to the heat transfer between the control volume and its surroundings, $A_{w}=-W_{cv}+P_{o}\left(V_{2}-V_{1}\right)$ is equal to the negative value of the work produced by the control volume $W_{cv}$ but in most cases, the control volume has a constant volume, therefore $A_{w}$ can be further simplified, and $I=T_{amb}.S_{gen}$ is the availability of destruction in the process. The flow availability is expressed as $A_{fi,o}=\sum_{i,o}m_{i.o}^{.}a_{fi,o}$. Thus, the general form in steady-state condition would become:

(64) $$0=A_{q}+A_{w}+A_{fi}-A_{fo}-I$$

In conventional economic analysis, a cost balance is usually formulated for the overall system operating at steady state as following:

(65) $$\sum_{out}C^{.}=\sum_{in}C^{.}+Z^{IC\&OM}$$

where $C^{.}$ the cost rate according to inlet and outlet streams, and $Z^{IC\&OM}$ is the capital investment and operating and maintenance costs. In exergy costing a cost is associated with each exergy stream. Thus, for inlet and outlet streams of matter with associated rates of exergy transfer $E_{i,o}^{.}$, power $W^{.}$, and the exergy transfer rate associated with heat transfer Equation $E_{q}^{.}$, can write as follows:

(66) $$C_{i,o}^{.}=c_{i,o}E_{i,o}^{.}$$

(67) $$C_{w}^{.}=c_{w}W^{.}$$

(68) $$C_{q}^{.}=c_{q}E_{q}^{.}$$

where $c_{i,o},w,q$ denote average costs per unit of exergy in (USD/Kj) for inlet (*i*), outlet (*o*), power (*w*), and energy (*q*) respectively. For hourly costs estimation, the following correlations are considered:

For cost analysis, the amortization factor $A_{f}$ is estimated based on the following relation:

(69) $$A_{f}=\frac{i.{\left(1+i\right)}^{LTp}}{{\left(1+i\right)}^{LTp}-1}$$

Collector investment cost $IC_{col}\;\left(\mathrm{USD}\right)$ is calculated based on the following area correlation:

(70) $$IC_{col}=150\times A_{col}^{0.95}$$

operating and maintenance cost $OMC_{col}$ is then calculated (USD):

(71) $$OMC_{col}=0.15\times IC_{col}$$

Total annual cost $TAC_{col}$ (USD/y) is then calculated based on operating and maintenance cost and investment cost parameters as following:

(72) $$TAC_{col}=\left(IC_{col}+OMC_{col}\right)\times A_{f}$$

hourly costs are calculated $Z_{col}$ (USD/h):

(73) $$Z_{col}=\frac{TAC_{col}}{OH\times 365}$$

Flashing tank investment cost $IC_{fsh}$ (USD) is calculated based on the following total tank volume $Vol_{fst}$ correlation:

(74) $$IC_{fsh}=\frac{Vol_{fst}\times 6.3e3}{3.8}$$

The total annual cost $TAC_{fsh}$ USD/y is then calculated:

(75) $$TAC_{fsh}=IC_{fsh}\times A_{f}$$

hourly costs are calculated $Z_{fsh}$ (USD/h):

(76) $$Z_{fsh}=\frac{TAC_{fsh}}{OH\times 365}$$

Absorption cycle investment cost $IC_{aac}\;\left(\mathrm{USD}\right),$ is calculated based on the following total area correlation:

(77) $$IC_{aac}=150\times A_{aac}^{0.8}$$

Total annual cost $TAC_{aac}$ (USD/y) is then calculated:

(78) $$TAC_{aac}=IC_{aac}\times A_{f},$$

hourly costs are calculated $Z_{aac}$ (USD/h):

(79) $$Z_{aac}=\frac{TAC_{aac}}{OH\times 365}$$

Pump investment cost $IC_{p}\;\left(\mathrm{USD}\right)$ is calculated based on the following pump power correlation:

(80) $$IC_{p}=3500\times W_{p}^{0.47}$$

Total annual cost $TAC_{p}$ (USD/y) is then calculated:

(81) $$TAC_{p}=IC_{p}\times A_{f}$$

hourly costs are calculated $Z_{p}$ (USD/h):

(82) $$Z_{p}=\frac{TAC_{p}}{OH\times 365}$$

Total hourly costs $Z_{tot}$ (USD/hr) is then calculated based on all parameters as following:

(83) $$Z_{tot}=Z_{col}+Z_{fsh}+Z_{aac}+Z_{p}$$

Total Plant Costs TPC (USD/y)is also calculated based on the total annual costs for all unit:

(84) $$TPC=TAC_{col}+TAC_{fsh}+TAC_{aac}+TAC_{p}$$

The total thermo-economic equation is calculated based on the cost and exergy stream through the proposed cycles (USD/GJ):

(85) $$c_{p}=1000\times \left(\frac{\left(W_{tot}\times c_{w}\right)+\left(\frac{Z_{tot}}{3600}\right)}{Ex_{fo}}\right)$$

where, $c_{p}$ is the thermo-economic product cost (USD/GJ), $c_{w}$ is the power cost in (USD/kWh) (~0.065), and $W_{tot}$ is the total cycle power (kW), $Ex_{fo}$ is the exergy stream outlet from the system to the user (kW).

For performance calculations, the COP is calculated based on evaporator $Q_{e}$ and generator $Q_{g}$ thermal powers:

(86) $$COP=\frac{Q_{e}}{Q_{g}}$$

where the Max COP is found as:

(87) $$COP_{max}=\frac{\left(T_{e}+273.15\right)\times \left(T_{g}-T_{a}\right)}{\left(T_{g}+273.15\right)\times \left(T_{c}-T_{e}\right)}$$

and the relative performance ratio RPR could be then estimated as:

(88) $$RPR=\frac{COP}{COP_{max}}$$

The values of the design variables in Table 1 will be used in this work. The equations comprising the mathematical model of the combined system are utilized in MATLAB software, and the results are presented hereafter.

## 4. Results and Comments

As illustrated earlier, the proposed cycles for the current study are two cycles. Therefore, it is very important to optimize the absorption air conditioning AAC cycle before the attachment of solar collectors. Such optimization would reduce the design aspects, such as area. Meanwhile, lowering of the cost values and the thermo-economic product cost goes to the end-user. For such optimization purposes, different operating conditions have been examined in this section. The optimization processes addressed the effect of different operating conditions on COP, COP_max_, Exergy destruction rate, mass flow rates, and design aspects such as areas and volume.

### 4.1. Optimization of NH_3_-H_2_O Cycle

#### 4.1.1. Absorber Temperature Effect

**Figure 2:**
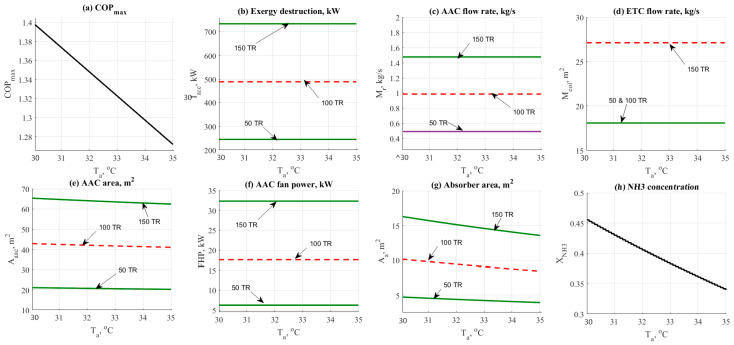
NH_3_-H_2_O data results based on the effect of absorber temperature parameter.

The effect of changing absorber temperature on the system performance parameters is shown in Figure 2 such as COP_max_, exergy destruction rate, mass flow rates, and design aspects. The data has been obtained at different cooling loads (50–150 TR), and different values of absorber operating temperature (30–35 °C). The effect of absorber temperature on the COP_max_ of the absorption cycle is illustrated in Figure 2a. Increasing the absorber temperature would cause a notable decrease in the COP_max_. This was happened because of the following relation between the absorber temperature and COP_max_ ($COP_{max}=\frac{\left(T_{e}+273.15\right)\times \left(T_{g}-T_{a}\right)}{\left(T_{g}+273.15\right)\times \left(T_{c}-T_{e}\right)}$). In general, the COP_max_ was in the relatively higher range of 1.2–1.4. Figure 2b shows that there is no significant change in the exergy destruction rate. The same behavior was noticed on the total mass flow rate as shown in Figure 2c. The mass flow rate has not been changed vs. the increasing absorber temperature.

The effect of absorber temperature on the flow rate is not significantly high while comparing against the refrigerant load. The same behavior was noticed in Figure 2d. Absorber temperature has no effect on the ETC mass flow rate. A minor reduction in the AAC area has been noticed in Figure 2e. For instance, the total AAC area has been decreased from 65 down to 62 m^2^ at 150 TR load. Figure 2f shows that there is no effect on the evaporator fan cooler unit. The effect would happen in case of the change in evaporator temperature. Figure 2g reflected the change in the absorber area by the cause of absorber temperature. The area has been decreased significantly. Figure 2h shows that by increasing the absorber temperature the NH_3_ concentration has been decreased with no effect of the cooling load.

#### 4.1.2. Condenser Temperature Effect

**Figure 3:**
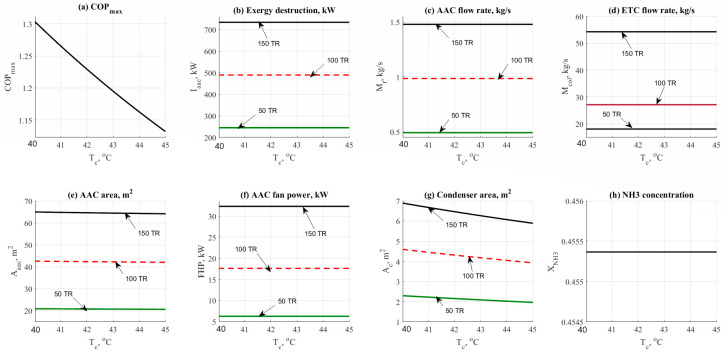
NH_3_-H_2_O data results based on the effect of condenser temperature parameter.

Figure 3 shows the data result based on the effect of condenser temperature on the other design parameters such as COP_max_, exergy destruction rate, mass flow rates, and design aspects. Data has been obtained at different refrigerant loads (50–150 TR), and at different values of condenser operating temperature (40–45 °C). Figure 3a shows the effect of condenser temperature on the COP_max_. Increasing the condenser temperature would cause a notable decrease in the COP_max_. At the same time, increasing the load has no effect on the COP_max_ as noticed on the same figure. Figure 3b shows that there is no significant change in the exergy destruction rate. The same behavior was noticed on the total mass flow rate as shown in Figure 3c; the mass flow rate has not been changed vs. the increase of condenser temperature.

The effect of condenser temperature on the flow rate is not significantly high while comparing against the refrigerant load. The same behavior was noticed in Figure 3d. Condenser temperature has no effect on the ETC mass flow rate too. A minor reduction in the AAC area has been noticed in Figure 3e. For instance, the total AAC area has been decreased from 65 m^2^ down to 64 m^2^ at 150 TR load. Figure 3f shows that there is no effect on the evaporator fan cooler unit. The effect would be happened in case of the change in evaporator temperature or based on the temperature difference between the room temperature and the user target temperature. Figure 3g reflected the change in the condenser area by the cause of condenser temperature. The area has been decreased significantly causing a minor decrease in the total AAC area. The condenser area was ranged between 2 and 7 m^2^ at 50 TR and 150 TR respectively. Figure 3h shows that by increasing the condenser temperature the NH_3_ concentration has not been changed and it remained constant at 45% concentration.

#### 4.1.3. Evaporator Temperature Effect

**Figure 4:**
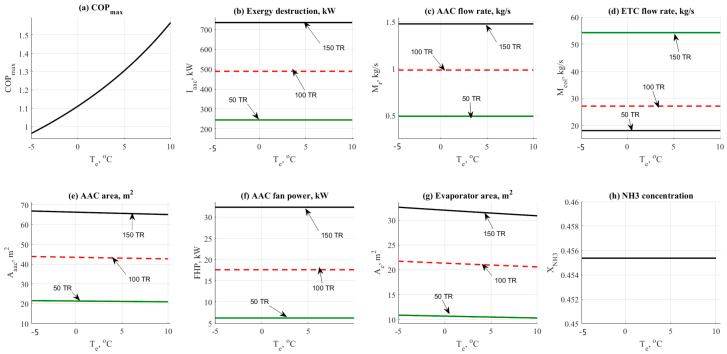
NH_3_-H_2_O data results based on the effect of evaporator temperature parameter.

Figure 4 shows the data result based on the effect of evaporator temperature on the other design parameters such as COP_max_, exergy destruction rate, mass flow rates, and design aspects. Data has been obtained at different refrigerant loads (50–150 TR) and at different values of evaporator operating temperature (−5 to 10 °C). Figure 4a shows the effect of the evaporator temperature on the COP_max_. Increasing the evaporator temperature would cause a notable increase in the COP_max_. At the same time, increasing the load has no effect on the COP_max_ as noticed on the same figure. Figure 4b shows that there is no significant change in the exergy destruction rate caused by the evaporator temperature. The same behavior was noticed on the total mass flow rate as shown in Figure 4c. The mass flow rate has not been changed vs. the increase of evaporator temperature. The effect of evaporator temperature on the flow rate is not significantly high while comparing against the refrigerant load. The same behavior was noticed in Figure 4d. Evaporator temperature has no effect on the ETC mass flow rate too. A minor reduction in the AAC area has been noticed in Figure 4e. For instance, the total AAC area has been decreased from 65 down to 64 m^2^ at 150 TR load. Figure 4f shows that there is no effect on the evaporator fan cooler unit. The effect would be happened in case of the change in evaporator temperature or based on the temperature difference between the room temperature and the user target temperature. Figure 4g reflected the change in the evaporator area by the cause of the evaporator temperature. The area has been decreased significantly causing a minor decrease in the total AAC area. The evaporator area was ranged between 10 and 35 m^2^ at 50 and 150 TR respectively Figure 4h shows that by increasing the evaporator temperature the NH_3_ concentration has not been changed and it remained constant at 45% concentration.

#### 4.1.4. Generator Temperature Effect

**Figure 5:**
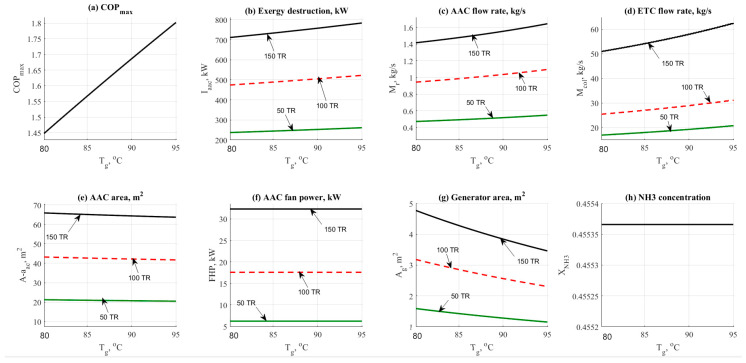
NH_3_-H_2_O data results based on the effect of generator temperature parameter.

The generator operating temperature has a great influence on this cycle. Figure 5 shows the data result based on the effect of generator temperature on the other design parameters such as COP_max_, exergy destruction rate, mass flow rates, and design aspects. Data has been obtained at different refrigerant loads (50–150 TR) and different values of the generator operating temperature (80 to 95 °C). Figure 5a shows the effect of the generator temperature on the COP_max_. Increasing the generator temperature would cause a notable increase in the COP_max_ from 1.45 to 1.8. The driving temperature depends on the solar energy capturing technology that can be adopted and efficiently available. At the same time as shown in Figure 5b that the exergy destruction has also increased as a normal reflection to the generator temperature changing. The same behavior was noticed on the total mass flow rate as shown in Figure 5c. The mass flow rate has been changed to be increasing vs. the increasing generator temperature.

The effect of generator temperature on the flow rate is significantly high. The same behavior was noticed in Figure 5d. Generator temperature has a remarkable effect on the ETC mass flow rate too. A minor reduction in the total AAC area has been noticed in Figure 5e. For instance, the total AAC area has been decreased from 65 down to 62 m^2^ at 150 TR load. Figure 5f shows that there is no effect on the generator fan cooler unit. Figure 5g reflected the change in the generator area by the cause of the generator temperature. The area has been remarkably decreased causing a minor decrease in the total AAC area. The generator area was ranged between 1.5 and 5 m^2^ at 50 and 150 TR respectively Figure 5h shows that by increasing the generator temperature the NH_3_ concentration has not been changed and it remained constant at 45% concentration.

#### 4.1.5. Solar Field Top Temperature Effect

**Figure 6:**
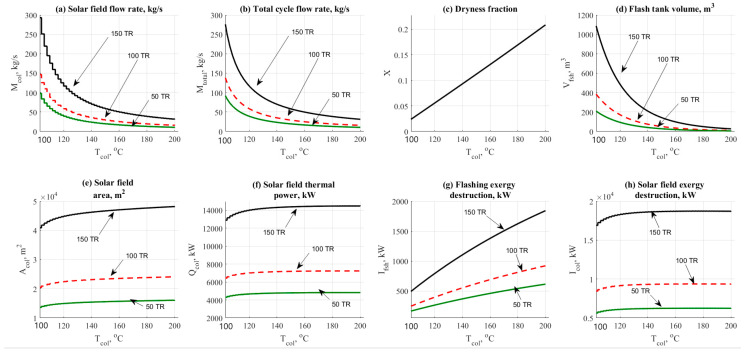
NH_3_-H_2_O data results based on the effect of ETC outlet top temperature.

**Figure 7:**
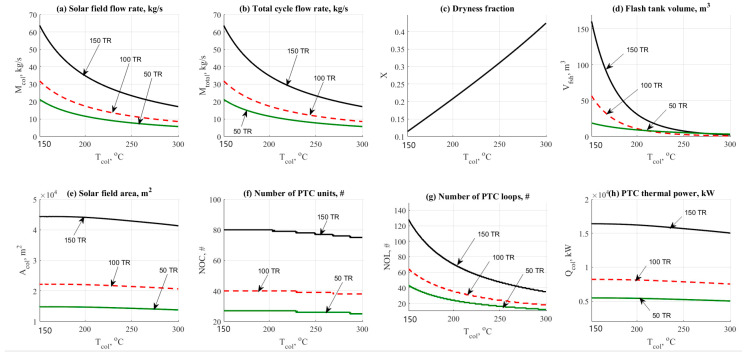
NH_3_-H_2_O data results based on the effect of PTC outlet top temperature.

Solar field top temperature is considered a very important parameter in this cycle. Figure 6 and Figure 7 show the variations on the performance and design parameters for both operational cases ETC and PTC. For ETC, the temperature range was 110 to 200 °C. The temperature range for PTC was 150 to 300 °C. Figure 6a shows the effect of ETC top temperature on the solar field mass flow rate.

The figure shows that increasing the top temperature would reduce the total mass flow rate as a normal result extracted from the energy balance across the solar field. The same behavior was also noticed in Figure 7a which represents the PTC operation. However, PTC conceded lower flow rates which means lower costs. The difference is considered huge if we compared between 50 to 300 kg/s @ 150 TR for the ETC and 15 to 65 kg/s @ 150 TR for the PTC. The same behavior was also noticed for both collector’s type related to the total cycle flow rate as shown in Figure 6b and Figure 7b.

Figure 6c and Figure 7c also reflects the advantage of using PTC rather than ETC. The figure shows that the dryness fraction was in increasing mode by the increase of the solar filed operating temperature. ETC gives a range of 10% to 20% of generating steam while the PTC gave 20% to 40% which is considered attractive and reasonable. For both cases, flash tank volume has been presented in Figure 6d and Figure 7d. The difference is quite clear with an advantage to the PTC.

Increasing the top solar field temperature would decrease the flashing tank volume. However, ECT will need larger tanks because of lower dryness fraction in comparison against the PTC operation. Figure 6e and Figure 7e shows the effect of solar temperature on the solar field design area. The changes in Figure 6e and Figure 7e were not massive; however, lower area, i.e., lower costs and control are recorded for the PTC as expected.

For instance, at 150 TR, ETC will consume about 45,000 to 50,000 m^2^. However, the PTC will consume about 45,000 down to 40,000 m^2^ under the same operating conditions. For ETC, Figure 6f–h represents the effect on the thermal power, kW, flashing, and solar field exergy destruction rates, kW. Increasing the operating temperature would increase thermal power and exergy destruction rates as well.

Figure 7f,g shows that increasing the PTC temperature would decrease the number of units and the number of loops as well. The number of loops parameter is affected by the total PTC area required for the load. Figure 7h shows the effect on PTC thermal power. The figure shows a decreasing behavior vs. the increasing of the top solar field temperature. Generally, increasing the load from the AAC unit would request more solar field areas leading to the increase of mass flow rate and exergy destruction rates.

A value of 150~200 °C and 250~300 °C would be recommended in this study for ETC and PTC solar collectors respectively. Based on the currently obtained results, it is quite interesting to assign the operating temperature as follows:

➢ *T_a_* = 35 °C.
➢ *T_c_* = 43 °C.
➢ *T_e_* = 7~10 °C.
➢ *T_g_* = 85~90 °C.
➢ ETC *T_high_* = 150~200 °C.
➢ PTC *T_high_* = 250~300 °C.

#### 4.1.6. Cooling Load Effect

**Figure 8:**
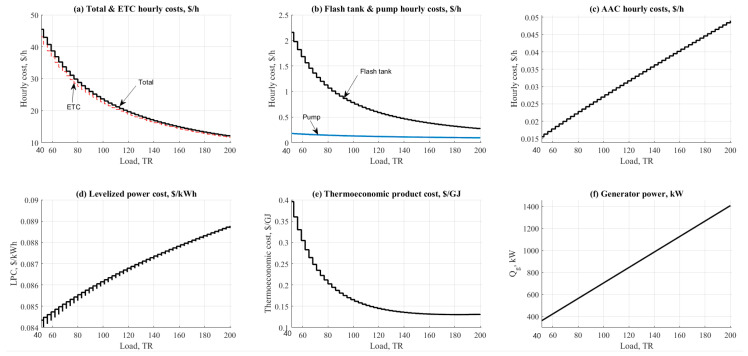
ETC-NH_3_-H_2_O data results based on the effect of cooling load.

**Figure 9:**
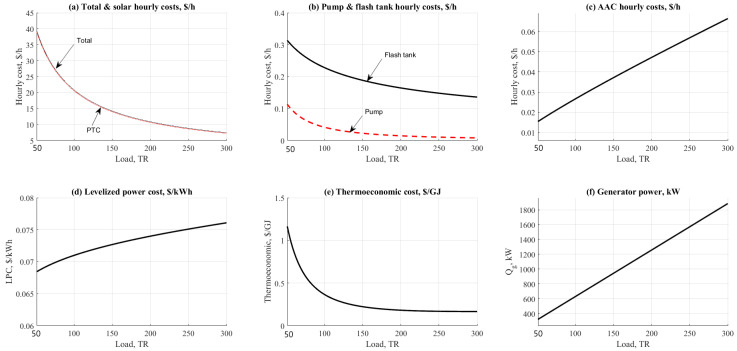
PTC-NH_3_-H_2_O data results based on the effect of cooling load.

The cooling load effect on ETC/NH_3_-H_2_O and PTC/NH_3_-H_2_O cycles are shown in Figure 8 and Figure 9. Both figures addressed the effect on hourly costs, Levelized power cost, and thermo-economic product cost. As expected, the behavior on the figure was in varying mode due to the load and energy demand for all units. Solar collectors recorded the highest values of hourly costs among the other units as shown in Figure 8a and Figure 9a.

However, PTC was recorded lower by 8% against the ETC related to the hourly costs parameter. The main reason for this was referring to the increasing in collector operating temperature (250 vs. 150 °C). Increasing the collector temperature will generate more steam for the AAC unit. AAC, pumps, and flashing tanks are shown in Figure 8b,c and Figure 9b. It was clear on the figures that the flashing tank is recorded higher than the pump and the AAC unit based on the total tank volume. PTC operation was recorded lower in flashing tank hourly cost because it has lower tank volume 0.2–0.3 $/h vs. USD 0.5–2.2/h. Figure 8d and Figure 9d show the effect of cooling load on the levelized power cost, USD /kWh.

The results are nearly the same based on the close results between the two configurations related to the pumping unit. The levelized power cost was ranged between USD 0.07/kWh to USD 0.08/kWh for PTC, ETC respectively. The same close behavior was noticed while comparing related to the thermo-economic product cost. Results were centralized 0.1 to USD 1/GJ for both configurations with a minor advantage to the ETC (Figure 8e and Figure 9e). As anticipated, increasing the load would increase the cost of exergy. Solar PTC and/or ETC are considered the main cause of such an effect related to the large area needed to cover on the load. Figure 8f and Figure 9f show the effect on the generator thermal power which is recorded increasing vs. the increase of cooling load.

### 4.2. Case Study Results

The case study presented in this section compares between two configurations at a specific load point. The case study was about a sports arena which is located in Baghdad, Iraq. The arena is a USD 14 million, 3000-seat indoor sports facility is focused around basketball, volleyball, and athletics. The whole project will be centrally air-conditioned by a 700–800 kW solar absorption cycle. The evaporator will be designed to work between 7 and 12 °C. Table 2 shows the data results for two configurations in case of using the solar absorption cycle. A 200 TR cooling load has been selected as an example of such a comparison. Environmental operating conditions are fixed at a specific value for simplicity. From solar field results, PTC/NH_3_-H_2_O resulted in the lowest in the area needed, and it is quite important to reduce the needed area. ETC/NH_3_-H_2_O comes next. For design aspects such as flashing tank design, it was clear the operation of PTC/NH_3_-H_2_O gives the lowest results which were the most favorable, at 3.8 m^3^, followed by ETC/NH_3_-H_2_O with 7.8 m^3^. The same behavior was also noticed related to the dryness fraction. The same behavior is also noticed related to the exergy destruction rate. The results reveal that PTC is considered an advantage to the cycle vs. the ETC operation. PTC/NH_3_-H_2_O recorded lower in the absorber area with 21 m^2^. Minimum driving steam was recorded by PTC/NH_3_-H_2_O (1.8 kg/s) which lead to low flashing tank volume and lower solar field area. COP was found remarkable related to PTC/NH_3_-H_2_O cycle. For hourly costs, PTC/NH_3_-H_2_O is noticed as the lowest among the rest. By achieving USD11.3/h, PTC/NH_3_-H_2_O is considered the best option for this case study. Lower hourly costs for the solar field is considered the vital term to judge the system cost. Thermo-economic cost is nearly the same for two configurations within the range of USD 0.14–0.15/GJ with an advantage to PTC/NH_3_-H_2_O configurations.

## 5. Conclusions

A two configurations of solar-assisted NH_3_-H_2_O absorption refrigeration system was proposed, modeled, and simulated in this paper. A Flash evaporation tank has been used as a steam generation unit between the solar part and absortion chiller. A parametric study on the effect of the temperature of the absorption cycle component on the performance of the system is carried out and the results are presented. In addition, energy, exergy and cost analyses are presented in this work. According to the analysis results, the following conclusions can be drawn:

▪ Design aspects, such as solar area and flashing tank volume were found to have a great influence on the cycle cost.
▪ Increasing the cooling load increases the required solar field area and the flow rate of the heat transfer fluid.
▪ Optimization of the operating conditions, such as temperatures, has been performed for two configurations. The following values of operating conditions are considered the best, related to design aspects, COP, exergy destruction rate, and cost:
  ○ *T_a_* = 35 °C.
  ○ *T_c_* = 43 °C.
  ○ *T_e_* = 7~10 °C.
  ○ *T_g_* = 85~90 °C.
  ○ ETC *T_high_* = 150~200 °C.
  ○ PTC *T_high_* = 250~300 °C.
▪ A case study is presented in a sports arena located in Baghdad, Iraq, for which the needed cooling load was in the range of 700 to 800 kW. PTC/NH_3_-H_2_O was recorded the best based on design and hourly costs. The required solar area was in the range of 2000–2500 m^2^. While the total hourly costs were in the range of USD 11.3–12.6/h which is quite attractive.
▪ PTC/NH_3_-H_2_O gives the lowest values related to exergy destruction rates for all units. As expected, the solar filed would harvest a larger amount of exergy destruction rates for two configurations due to the large area and mass flow rate effect. The PTC/NH_3_-H_2_O exergy destruction rate results are in the range of 4600–5000 kW.
▪ PTC/NH_3_-H_2_O gives the lowest value of flashing tank design aspects such as width, 1.343 m, height, 2.743 m, and volume, 3.883 m^3^. ETC/NH_3_-H_2_O comes next with a total flashing tank equal to ~7.276 m^3^.
▪ It is quite clear that PTC/NH_3_-H_2_O followed by ETC/NH_3_-H_2_O have a remarkable result according to the terms of energy, design, and cost. Generally, the PTC system is considered the best choice for the NH_3_-H_2_O solar cooling system.

## Figures and Tables

**Table 1:** Data assumptions for the evacuated tube collector/parabolic trough collector (ETC/PTC)/NH_3_-H_2_O configuration.

Unit Process	Assigned Data	Calculated Data
**Absorption air-conditioning cycle (AAC): (ETC/PTC)/NH_3_-H_2_O**	✓ Solar radiation: 500 W/m^2^ ✓ Ambient temperature: 25 °C ✓ Average relative humidity: 15% ✓ ETC top temperature: 100–200 °C ✓ PTC top temperature: 200–300 °C ✓ Absorber temperature: 30–35 °C ✓ Generator temperature: 80–90 °C ✓ Condenser temperature: 40–45 °C ✓ Hot air temperature: 35 °C ✓ Target cooled air temperature: 20 °C ✓ Evaporator temperature: 5–10 °C ✓ Cooling load: 14–57 kW (50–200 TR) ✓ Condenser effectiveness: 80% ✓ Fan system efficiency: 85% ✓ Pumping system efficiency: 75% ✓ Plant life time: 20 year ✓ Interest rate: 5% ✓ Load factor: 90% ✓ Specific electric power cost: 0.065 $/kWh ✓ Water steam is the solar field working fluid	**Solar Field:** ➢ Solar field top pressure, bar ➢ Solar field pressure loss, bar ➢ Total solar field area, m^2^ ➢ Solar field thermal load, kW ➢ Number of solar collectors, # ➢ Solar field mass flow rate, kg/s ➢ Solar field inlet temperature, °C ➢ Efficiency, % ➢ Exergy destruction, kW **Flash Tank:** ➢ * Flash tank design data ➢ Total mass flow rate, kg/s ➢ Dryness fraction, % ➢ Flash tank water flow rate, kg/s ➢ Steam flow rate, kg/s ➢ Exergy destruction, kW **AAC Unit:** ➢ Weak and strong solutions, kg/s ➢ * Design data ➢ Thermal power, kW ➢ Total cycle flow rate, kg/s ➢ Generator power, kW ➢ Cooling fan power, kW ➢ COP ➢ COPmax ➢ Relative performance ➢ Exergy destruction, kW **Pump:** ➢ Power, kW ➢ Outlet temperature, °C ➢ Exergy destruction, kW **Cost and Performance:** ➢ Units hourly costs, $/h ➢ Total hourly costs, $/h ➢ Total power, kW ➢ LPC, $/kWh ➢ Thermo-economic cost, $/GJ ➢ Total exergy destruction rate, kW
**Notes:**	▪ Data are run out based on steady-state operating conditions. ▪ Ambient temperature is fixed as 25 °C for all process runs. ▪ Solar radiation is fixed at 500 W/m^2^. ▪ * Design data means area, length, width, etc.	

**Table 2:** Data results based on 200 TR load case study.

**Solar radiation, W/m^2^**	500	
***T_amb_*, °C**	25	
***T_a_*, *T_e_*, *T_g_*, *T_c_*, *T_col_*, °C**	30, 10, 90, 40, 175	30, 10, 90, 40, 250
**Load, TR**	200	
**Target cooled air, °C**	20	
**Interest rate, %**	5	
**Load factor, %**	95	
**Plant life time, yr**	20	
**Electric cost, $/kWh**	0.065	
**Fans efficiency, %**	80~85	
**Pumps efficiency, %**	75	
**Configuration:**	**ETC/NH_3_-H_2_O**	**PTC/NH_3_-H_2_O**
**Solar field:**		
**Total solar field area, m^2^**	1.188e4	1.102e4
**Solar thermal power, kW**	3612	4051
**Inlet temperature, °C**	91.82	92.04
**Mass flow rate, kg/s**	10.17	8.727
**Inlet exergy, kW**	5646	4217
**Exergy destruction, kW**	4683	5143
**Flash tank:**		
**Height/Width, m**	3.381/1.655	2.743/1.343
**Volume, m^3^**	7.276	3.883
**Total flow rate, kg/s**	10.17	8.727
**Water content, kg/s**	8.551	6.91
**Dryness fraction**	0.1595	0.208
**Exergy destruction, kW**	391.1	515.6
**AAC unit:**		
***Q_a_, kW***	1251	1153
***A_a_, m^2^***	23.37	21.3
***M_str_, kg/s***	1.428	1.428
***M_wk_, kg/s***	0.778	0.778
***X_hex-NH3_***	0.4554	0.4554
***A_hex_, m^2^***	7.837	6.73
***Q_g_, kW***	1407	1256
***A_g_, m^2^***	5.123	4.574
**Driving steam flow, kg/s**	2.07	1.848
***Q_c_, kW***	694.9	694.9
***A_c_, m^2^***	9.1	9.1
***M_r_, kg/s***	0.6505	0.6505
***Q_e_, kW***	703.4	703.4
***A_e_, m^2^***	41.15	41.15
***FHP, kW***	70.64	70.64
***M_air_, kg/s***	119.5~120	119.5~120
***COP/COP_max_***	0.5/1.559	0.5/1.559
***RPR***	0.3206	0.3591
**Exergy destruction, kW**	1010	977.3
**Fan exergy destruction, kW**	164	164
**Pump unit:**		
***W_p_, kW***	12.63	18.97~19
**Exergy destruction, kW**	64.7	62.25
**Cost and Thermo-economics:**		
***Z_col_,*** USD/**h**	11.75	10.93
***Z_fsh_,*** USD/**h**	0.11	0.06
***Z_aac_,*** USD/**h**	0.0487	0.04716
***Z_p+f_,*** USD/**h**	0.256	0.2652
***Z_tot_,*** USD/**h**	12.16	11.3
***LPC,*** USD/**h**	0.089	0.0744
***cp,*** USD/***GJ***	0.144	0.1554

## Nomenclature

*A*	Availability, kW, Area, m^2^
*A_t_*	Tube cross-sectional area, m^2^
*A_f_*	Amortization factor, 1/y
*AAC*	Absorption Air Conditioning
*C*	Thermo-economic cost stream, USD/kJ
*C_p_*	Specific heat capacity, kJ/kg °C @ constant pressure
*COP*	Coefficient of Performance
*D*	Diameter, m
*D_env_*	Collector glass envelope diameter, m
*E*	Exergy stream, kW
*ETC*	Evacuated Tube Collector
*f*	Function
*FHP*	Fan power, kW
*H*, *h*	Height, m, Enthalpy, kJ/kg
*H_dish_*	Dish parabola height, m
*I_s_*	Solar intensity, W/m^2^
*i*	Interest rate, %
*L*	Length, m
*L_m_*	Module length, m
*LMT*	Logarithmic Mean Temperature, °C
*LPC*	Levelized Power Cost, USD/kWh
*LTp*	Plant life time, y
*M*	Mass flow rate, kg/s
*NOT*	Number of Tubes
*NOC*	Number of Collectors
*NOL*	Number of Loops
*P*	Power, or Pressure, bar
Δ*P*	Pressure, bar
*Q*	Thermal power, kW
*RPR*	Relative Performance Ratio
*Re*	Raynold’s Number
*S*, *s*	Entropy, kJ/kg °C
*SHX*	Solution Heat Exchanger
*T*	Temperature, °C
*U*	Overall heat transfer coefficient, W/m^2^ °C
*V*, *Vol*	Volume, cm^3^
*v*	Velocity, m/s
*W*	Power, Work, kW
*W_c_*	Collector width, m
*X*	Concentration percentage, %
*Z*	Hourly cost, USD/h
***Subscripts***	
*_a, abs_*	Absorber
*_air_*	Air
*_amb_*	Ambient
*_a-hex_*	From absorber to heat exchanger stream
*_c_*	Condenser
*_c-evp_*	From condenser to evaporator stream
*_col_*	Collector
*_cw_*	Cooling water
*_e_*	Evaporator
*_etc_*	Evacuated tube collector
*_e-abs_*	From evaporator to absorber stream
*_f_*	Liquid phase
*_fan_*	Fan
*_fsh_*	Flashing tank
*_fst_*	Flashing steam
*_g_*	Generator, vapor phase
*_g-hex_*	From generator to heat exchanger stream
*_i_*	Inlet
*_loop_*	Loop
*_o_*	Out
*_p_*	Pump
*_ptc_*	Parabolic trough collector
*p_i,o_*	Pump inlet and outlet
*_q_*	Heat
*_r_*	Refrigerant
*_s_*	Steam
*_st_*	Steam
*_str_*	Strong
*_t_*	Turbine, total
*_w_*	Water
*_wk_*	Weak solution
***Greek***	
*η*	Efficiency, %
*ρ*	Density, kg/m^3^
*µ*	Dynamic viscosity, Pa.s
